# Decrease of Markers Related to Bone Erosion in Serum of Patients with Musculoskeletal Disorders after Serial Low-Dose Radon Spa Therapy

**DOI:** 10.3389/fimmu.2017.00882

**Published:** 2017-07-25

**Authors:** Aljona Cucu, Kateryna Shreder, Daniela Kraft, Paul Friedrich Rühle, Gerhart Klein, Gerhard Thiel, Benjamin Frey, Udo S. Gaipl, Claudia Fournier

**Affiliations:** ^1^GSI Helmholtz Center for Heavy Ion Research, Department of Biophysics, Darmstadt, Germany; ^2^Department of Radiation Oncology, Universitätklinikum Erlangen, Friedrich-Alexander-Universität Erlangen-Nürnberg, Erlangen, Germany; ^3^Association for Spa Research and Medical Practice for Cardiology, Bad Steben, Germany; ^4^Membrane Biophysics Group, Department of Biology, Technical University Darmstadt, Darmstadt, Germany

**Keywords:** chronic inflammatory diseases, degenerative musculoskeletal disorders, bone metabolism, osteoblasts, osteoclasts, adipokines, Treg/Th17 cells, radon spa treatment

## Abstract

Musculoskeletal disorders (MSDs) are the most frequent cause of disability in Europe. Reduced mobility and quality of life of the patients are often associated with pain due to chronic inflammation. The inflammatory process, accompanied by a destruction of the cartilage and bone tissue, is discussed as a result of (A) the infiltration of immune cells into the joints, (B) an altered homeostasis of the joint cavity (synovium) with a critical role of bone remodeling cells, and (C) release of inflammatory factors including adipokines in the arthritic joint. In addition to the classical medication, low-dose radiation therapy using photons or radon spa treatments has shown to reduce pain and improve the mobility of the patients. However, the cellular and molecular mechanisms of anti-inflammatory effects of radon are yet poorly understood. We analyzed blood and serum samples from 32 patients, suffering from MSDs, who had been treated in the radon spa in Bad Steben (Germany). Before and after therapy, we measured the levels of markers related to bone metabolism (collagen fragments type-1, cartilage oligomeric matrix protein, receptor activator of NFκB ligand, and osteoprotegerin) in the serum of patients. In addition, adipokines related to inflammation (visfatin, leptin, resistin, and adiponectin) were analyzed. Some of these factors are known to correlate with disease activity. Since T cells play an important role in the progression of the disease, we further analyzed in blood samples the frequency of pro- and anti-inflammatory T cell subpopulations (CD4^+^IL17^+^ T cells and CD4^+^FoxP3^+^ regulatory T cells). Overall, we found a decrease of collagen fragments (CTX-I), indicating decreased bone resorption, presumably by osteoclasts, in the serum of MSD patients. We also observed reduced levels of visfatin and a consistent trend toward an increase of regulatory T cells in the peripheral blood, both indicating attenuation of inflammation. However, key proteins of bone metabolism were unchanged on a systemic level, suggesting that these factors act locally after radon spa therapy of patients with MSDs.

## Introduction

Musculoskeletal disorders (MSDs) affect large part of the population and can have multiple origins. Given this, MSDs represent the highest cause of physical disability (1). Reduced mobility and quality of life of the patients are often associated with pain due to destructive and inflammatory processes at the respective sites of the body (2, 3). A major fraction of patients with MSDs suffers from osteoarthritis (OA). The disease is elicited by an unbalanced load of bone and cartilage, which in turn is causing attrition, succeeded by a progressive inflammatory process. Inflammation may become chronic and is then accompanied by further erosion of cartilage and bone, but also with concurrent bone formation (osteophytes) (4). Even though bone and cartilage destruction occurs in rheumatoid arthritis (RA) too, the pathogenesis of this autoimmune disease is different; in the pathogenesis of RA, inflammation is the trigger and not the consequence of bone and cartilage destruction (5).

For the treatment of MSDs, non-steroidal anti-inflammatory drugs (NSAIDs), opioids, and corticosteroid injections are most commonly used (6). NSAIDs and opioids legitimate only temporary treatments of acute or chronic pain as they can have significant associated morbidity and do not lead to functional improvement (7–9). In addition to the classical pharmacological treatment with NSAIDs and physiotherapeutic exercises, low-dose radiation therapy (LDRT) or radon spa treatment is alternative or complementary therapies for MSDs (10–12). LDRT, which is applied in several fractions with total doses ranging from 3.0 to 6.0 Gy X-rays, is clinically employed for the treatment of local chronic inflammatory diseases (11). In radon spa treatment, the radioactive radon-gas evaporating from rocks is used; the estimations for the total effective doses range from 0.05 to 2 mSv. The treatment consists of serial baths or repeated visits in mountain galleries. Clinical studies suggest that radon exposure has analgesic, anti-inflammatory, and immune-modulating effects (13–19). However, the underlying cellular and molecular mechanisms are largely unknown.

The present study (RAD-ON01) with patients suffering from MSDs was conceived for investigating a putative anti-inflammatory effect of radon exposure on the immune and skeletal system. To elucidate cellular changes leading to the observed clinical benefits from radon exposure, we investigated the serum concentrations of markers related to bone metabolism, prominent inflammatory key players such as adipokines as well as changes in subpopulations of T cells.

In spite of differences in the pathogenesis of RA and OA, the destruction of cartilage and bone tissue is discussed in both cases as a result of several interconnected processes in arthritic joints, namely (A) an infiltration of immune cells into the joint, (B) an altered homeostasis of the joint cavity (synovium), (C) an imbalance of bone and cartilage remodeling cells, and (D) a release of inflammatory cytokines including adipokines (20–22). A consequence of the imbalance between residing cells with either catabolic or anabolic functions is an enhancement of cartilage degradation and bone erosion. Bone erosion is caused by an elevated resorbing activity of osteoclasts (OCs) (23), which can be indirectly detected by increased levels of collagen fragments (CTX-I); the latter are considered as a marker of cathepsin K-mediated bone collagen degradation (24). In the case of arthritic disease, it is reported that the ratios of released receptor activator of nuclear factor kappa B ligand (RANKL), the OC differentiation factor receptor activator of NFκB ligand, and osteoprotegerin (OPG) are altered, compared to healthy individuals (21). OPG is known to compete with RANKL for receptor binding and is thus counteracting the OC stimulating effect of RANKL.

A high abundance of inflammatory cells (T and B cells, macrophages) in the synovial fluid of arthritic patients has been reported (25, 26); the presence of these cells contributes to destructive processes in joints via cytokine release (e.g., RANKL, IL-6, IL-1β, or TNF-α) (27). These cytokines, also adipokines, have been identified as regulators of inflammation-related processes which can also affect synovium or bone cells (28, 29). Adipokines are typically released by adipocytes. Elevated levels of adipokines such as adiponectin, visfatin, resistin, and leptin were detected in serum and synovia of RA and OA patients (22, 30, 31). In patients with RA, a decrease of serum levels of adipokines has been shown after combined therapy with infliximab and corticosteroids (32) and after treatment with conventional synthetic disease modifying drugs (csDMARDS), which are also used in OA (33, 34).

The working hypothesis of the present study was that radon therapy for MSD patients may lead to (1) an inhibition of bone resorption, and/or bone formation, and an inhibition of cartilage attrition, depending on the stage of the disease and (2) a decrease in the serum levels of adipokines. To explore this, in MSD patients we measured serum levels of markers related to bone turnover, i.e., CTX-I, cartilage oligomeric matrix protein (COMP), OPG, and RANKL, as well as adipokines associated with the pathogenesis of RA and OA, i.e., visfatin, adiponectin, leptin, and resistin. As adipokines themselves were shown to stimulate and promote the proliferation and activity of T cells (35), and since subsets of T cells are playing a central role in severity or resolution of inflammation, we suspected (3) an altered ratio of anti-inflammatory Treg and inflammatory Th17 cells in the serum of the patients.

## Materials and Methods

### Study Design and Patients

We prospectively studied a subgroup of patients enrolled in the RAD-ON01 trial with chronic degenerative MSDs of spine and/or joints. In March 2013, 100 patients were treated in the certified health resort Staatsbad Bad Steben [Bavaria, Germany; details published in Ref. (36)]. The radon treatment consisted of a series of nine baths with duration of 20 min each over 3 weeks. Temperature (34°C) and humidity have been controlled. The activity of the radon containing baths was 600 or 1,200 Bq/L, the respective cumulative dose was estimated to be 0.3 mSv (12). The study was carried out in accordance with the recommendations of the ethical review committee of the Bavarian State Chamber of Physicians (Bayerische Landesärztekammer, Munich, Germany, ethical approval BLÄK #12131). All patients have granted their written informed consent. Patients were included in the RAD-ON01 study if they fulfilled the following criteria:

1. Age of at least 18 years (up to 75 years)
2. Chronic degenerative MSDs of spine and/or joints
3. Pain anamnesis of at least 1 year
4. Pain intensity [visual analog scale (VAS) >4]
5. Accessibility of the patients (living in close proximity to Bad Steben)
6. Patient’s willingness to cooperate
7. Patient clarification and agreement
8. No participation in other studies (3 months before and during RAD-ON01 study)

Pain parameters, i.e., individual pain perception was evaluated by questionnaires filled in by every patient during regular medical examinations, using VAS, ranging from 0 (no pain) to 10 (worst pain imaginable).

In this work, in total 32 patients have been analyzed, most of them (*n* = 29) suffering from chronic pain in spine and/or joints. The mean age of the patients was 62 years (range 41–75 years). The patients did not receive any treatment with anti-inflammatory drugs during or after radon therapy. The patients were followed up before and in regular intervals after the start of therapy (6, 12, 18, and 30 weeks after the first radon bath). Medical examination was performed to measure pain and vascular parameters. Peripheral blood was drawn at indicated time points, transported to our laboratory and analyzed within 24 h.

The availability of serum from the individual patients was variable. The results of measurements, which we performed in more than 32 patients, are shown in the supplement (Figures S2 and S3 in Supplementary Material). Measurements of additional factors of bone metabolism obtained in less than 32 patients are presented in Figure S1 in Supplementary Material. For the analysis of the number of Treg and Th17 cells (Figure 4), before (0 weeks) and after therapy (6 weeks), only three patients could be analyzed. Therefore, we additionally measured the number of Treg and Th17 cells in 11 healthy individuals who were not treated with radon. The data from patients and healthy donors are displayed separately as indicated in the legend of Figure 4.

### Flow Cytometric Analysis of Treg/Th17 Cell Populations

From the peripheral blood of the patients, mononuclear cells (PBMCs) were isolated with BD Vacutainer CPT cell preparation tubes (BD Biosciences, Heidelberg, Germany) according to the manufacturer’s instructions. Immediately after isolation of PBMCs, staining of Treg and Th17 cells was performed with the human Th17/Treg phenotyping Kit (BD Pharmingen, Heidelberg, Germany) according to manufacturer’s staining protocol. Briefly, cells were washed with PBS and stained with markers against CD4, IL-17, and FoxP3 (PerCP-Cy5.5-CD4, PE-IL17 and Alexa Fluor^®^ 647-FoxP3). Expression of cell surface or intracellular markers was assessed using a flow cytometer (FACS Canto II, Becton Dickinson, Heidelberg, Germany). A typical dot plot and the gating strategy are shown in Figure 4. The frequency of cells related to the total number of CD4^+^ cells was analyzed with FlowJo software: CD4^+^FoxP3^+^ cells were classified as Treg cells and CD4^+^IL17^+^ cells as Th17 cells.

### Serum Levels of Markers Related to Bone Remodeling and Adipokines

Peripheral blood was taken into serum tubes (SST II Advance, BD, #366468) and centrifuged with 1,800 × *g* for 10 min at room temperature. Serum aliquots were stored at −80°C. Markers of bone and cartilage metabolism, i.e., serum carboxy-terminal collagen crosslinks of type-I collagen (CTX-I), osteoprotegerin (OPG), and COMP were determined in aliquots of serum samples, using *in vitro* diagnostic applicable ELISA assays obtained from Immunodiagnostic Systems Ltd. (Frankfurt/Main, Germany) and Immunodiagnostics AG (Bensheim, Germany). Total soluble RANKL (sRANKL) was measured by sRANKL ELISA, purchased from BioVendor (Brno, Czech Republic). In addition, levels of adipokines were measured in serum samples. ELISA for adiponectin and leptin was purchased from TECOmedical (Basel, Switzerland); for visfatin and resistin from AdipoGen (Liestal, Switzerland). All measurements were carried out according to the manufacturer’s instructions. Duplicate measurements were performed for each patient and each time point investigated. The raw data of all measurements are shown in Table S1 in Supplementary Material.

### Statistical Analysis

Statistical analysis was performed with two-tailed *t*-test for paired or independent samples after checking for normal distribution of the data points with D’Agostino and Pearson test. For distributions deviating from normal distributions, statistical significance was calculated with Wilcoxon matched pairs signed rank test (Graph Pad Prism 6, Graph Pad Software, La Jolla, CA, USA). Probability values <0.05 were considered significant. Spearman’s correlation coefficient (*r*) was determined to analyze the relation between pain perception (VAS) and Visfatin and CTX-I, respectively.

## Results

### Serum Levels of Markers of Bone Remodeling

**Figure 1:**
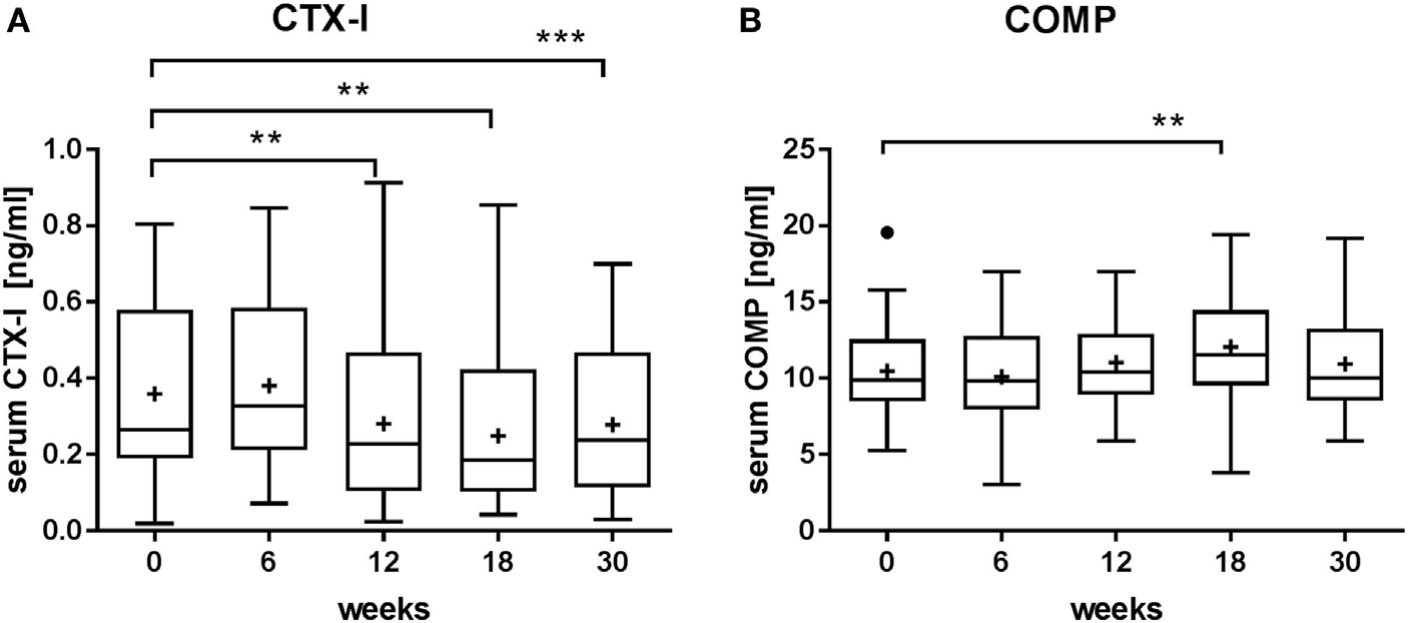
Effect of radon spa treatment on the levels of **(A)** collagen fragments type-1 (CTX-1) and **(B)** cartilage oligomeric matrix protein (COMP) in the serum of patients with musculoskeletal disorders (MSDs), measured at indicated times before (0 weeks) and after the onset of the therapy (6–30 weeks). Boxplots show the median, Tukey whiskers (median ± 1.5 times interquartile range), mean (+), and outliers (•). *N* = 32, **P ≤ 0.01, ***P ≤ 0.001, Wilcoxon matched-paired signed rank test.

To assess the effects of radon spa treatment on bone remodeling, we analyzed the levels of CTX-I, a marker used in clinical diagnostics, in the serum of MSD patients before and at indicated time points after radon spa treatment (Figure 1A). The levels of CTX-I dropped significantly 12 weeks after radon spa treatment and persisted at lower levels up to the end of the observation period (week 30). This result indicates decreased bone degradation as a consequence of radon spa treatment. A more detailed analysis of the data showed that the baseline levels were higher for female than male patients (Figure S4 in Supplementary Material). This is most likely due to postmenopausal changes related to the mean age of the female patients (62 years). The reduced CTX-I levels measured after the spa treatment were not accompanied by changes in the level of the OC inhibiting calcitonin, measured in a lower number of patients and presented in Figure S1D in Supplementary Material. To test for cartilage attrition, we assessed the serum levels of COMP, a glycoprotein belonging to the thrombospondin family (37). We did not find any significant changes between serum levels before and after therapy, except a slight increase for one time point (18 weeks) (Figure 1B).

**Figure 2:**
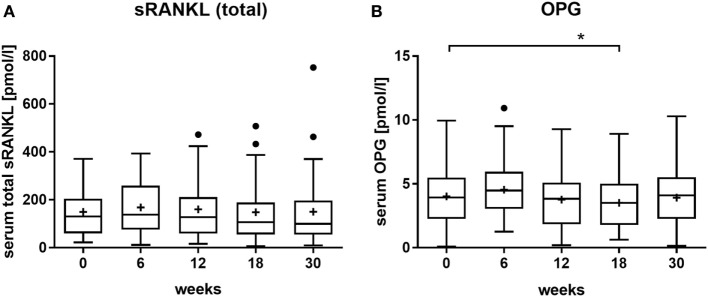
Effect of radon spa treatment on the levels of **(A)** total souble, receptor activator of nuclear factor kappa B ligand (sRANKL) and **(B)** osteoprotegerin (OPG) in the serum of patients with musculoskeletal disorders (MSDs), measured at indicated times before (0 weeks) and after the onset of the therapy (6–30 weeks). Boxplots show the median, Tukey whiskers (median ± 1.5 times interquartile range), mean (+), and outliers (•). *N* = 32, *P ≤ 0.5, two-tailed *t*-test.

Next, we analyzed the serum concentrations of the bone remodeling factors sRANKL (Figure 2A) and OPG (Figure 2B). The level of total sRANKL, which includes also the fraction of RANKL bound to OPG, remained unchanged after radon spa treatment. For OPG, a transient and significant decrease was detected, which occurred at one time point (18 weeks) after treatment.

In addition, we measured an OPG-unbound form of sRANKL (38), which we defined as “free” sRANKL (Figure S1A in Supplementary Material). No significant changes were observed, with only a trend discernible for a decrease at 12 and 30 weeks posttreatment was observed. Other factors indicating changes in the regulation of bone formation, such as BAP and osteocalcin (OCN), did not show any significantly modified levels after radon spa therapy (Figures S1B,C in Supplementary Material).

### Serum Concentration of Adipokines

**Figure 3:**
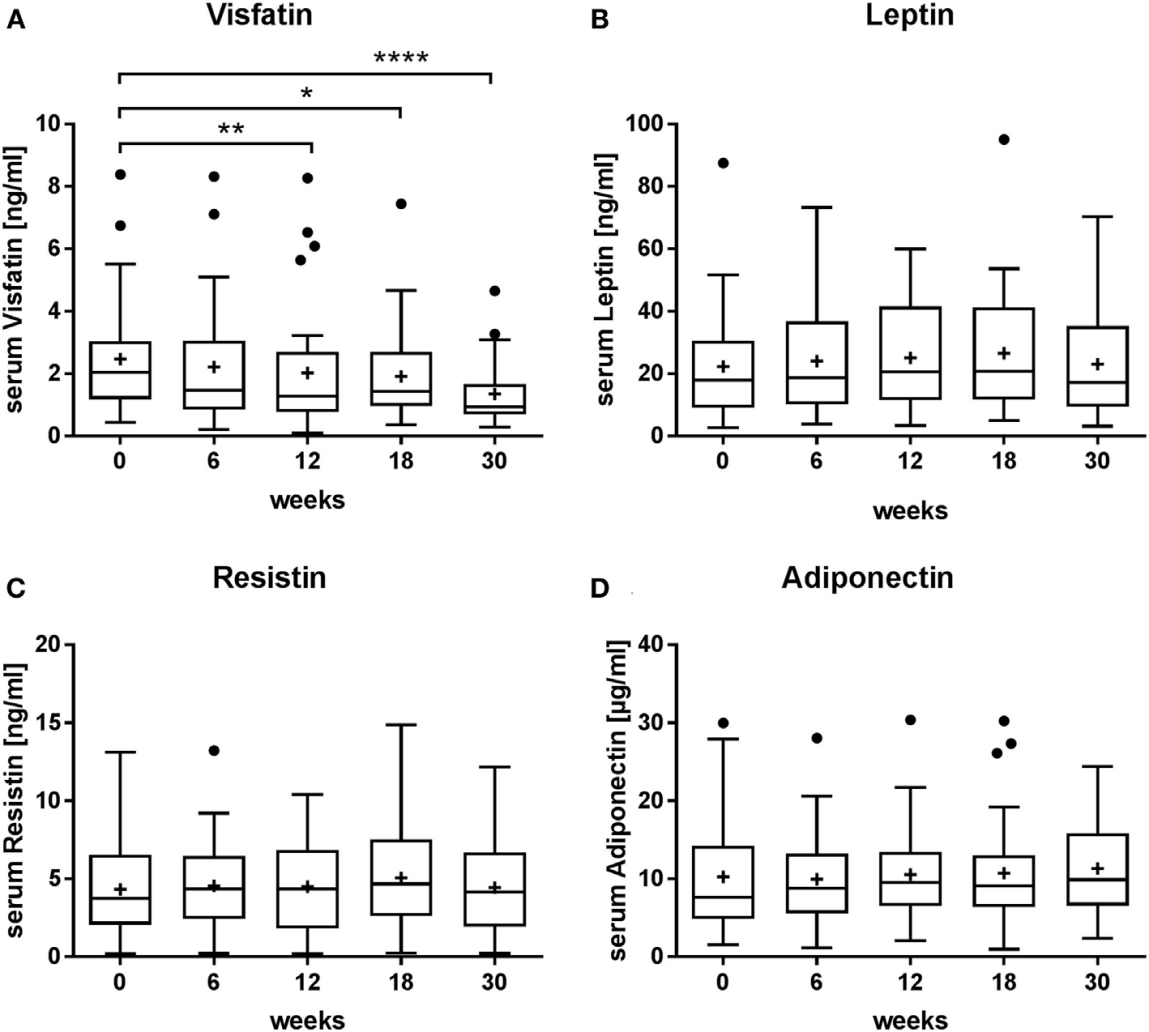
Effect of radon spa treatment on the levels of visfatin, leptin, resistin, and adiponectin in serum of patients with musculoskeletal disorders (MSDs). The concentration of appropriate adipokines was measured at the indicated weeks before (0 weeks) and after onset of the therapy (6–30 weeks). Boxplots show the median, Tukey whiskers (median ± 1.5 times interquartile range), mean (+), and outliers (•). *N* = 32, *P ≤ 0.05, **P ≤ 0.01, ****P ≤ 0.0001, Wilcoxon matched-paired signed rank test. **(A)** Visfatin. **(B)** Leptin. **(C)** Resistin. **(D)** Adiponectin.

To determine possible changes in the release of adipokines elicited by radon spa treatment, levels of selected adipokines have been measured in the serum of MSD patients. As shown in Figure 3A, the results revealed a significant decrease of visfatin levels after onset of the therapy, persisting at 30 weeks after start of the treatment. In contrast, the serum levels of leptin and resistin were not changed over the follow-up period (Figures 3B,C). Analysis of adiponectin levels showed no changes over 30 weeks as well (Figure 3D), although adiponectin levels of some patients were decreased after 6 and 12 weeks after therapy (not shown).

### Changed Frequencies of Treg and Th17 Populations in Peripheral Blood

**Figure 4:**
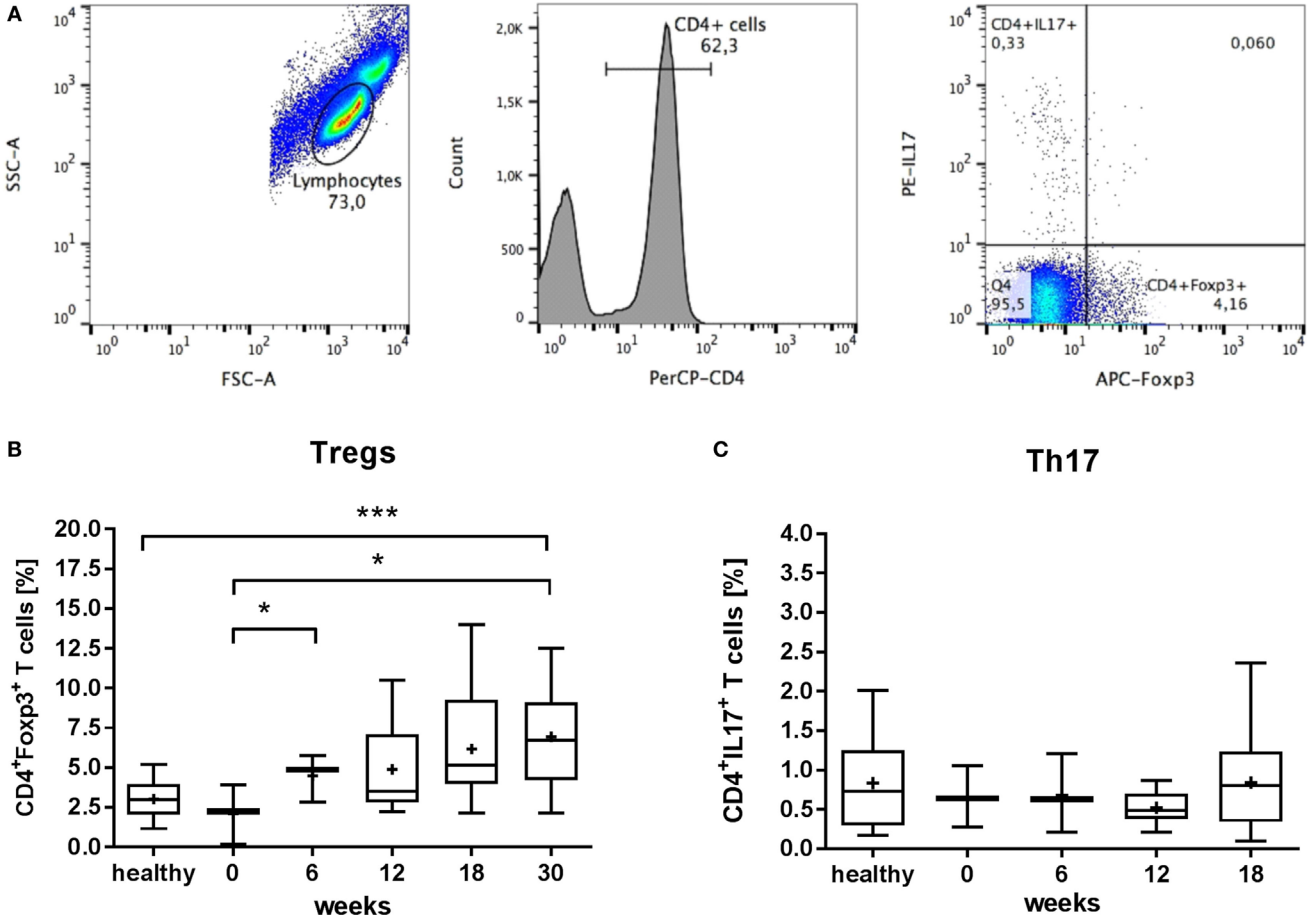
Subpopulations of in CD4^+^Foxp3^+^ T regulatory cells (Treg) and CD4^+^IL17^+^ T cells (Th17), related to the number of CD4^+^ T cells, isolated from the peripheral blood of musculoskeletal disorder (MSD) patients following radon spa treatment. **(A)** Representative dot plot and gating strategy for analysis of Treg/Th17 cells. Lymphocyte population was identified in the SSC/FSC dot plot. Only CD4^+^ cells were analyzed for PE-IL17 and APC-Foxp3 staining. **(B)** Percentage of CD4^+^Foxp3^+^ Treg cells in the peripheral blood of 3–20 patients (*N* = 3 for 0 and 6 weeks, *N* = 14 for 12 weeks, *N* = 20 for 18 weeks, and *N* = 16 for 30 weeks). Due to a limited number of samples available before treatment (0 weeks), data were compared with 11 healthy controls. **(C)** Percentage of CD4^+^IL17^+^ T cells in the peripheral blood of 3–20 patients (*N* = 3 for 0 and 6 weeks, *N* = 14 for 12 weeks, and *N* = 20 for 18 weeks), compared with 11 healthy controls. The samples at 30 weeks were lost due to technical problems during measurement. Boxplots show the median, Tukey whiskers (median ± 1.5 times interquartile range), mean (+), and outliers (•).*P ≤ 0.5, ***P ≤ 0.001, two-tailed *t*-test.

Using flow cytometry, we evaluated the frequencies of anti-inflammatory Treg cells and their opponents Th17 cells by intracellular staining of FoxP3 or IL-17, respectively. As shown in Figure 4, we observed an increasing fraction of FoxP3-positive Treg cells (2.1 to 6.9%, related to the total number of CD4^+^ cells) in patients following therapy (30 weeks) compared to the frequencies before treatment and those found in healthy donors (*P* = 0.001). However, the frequencies of IL17^+^ Th17 cells in the peripheral blood of patients were unchanged.

Taken together, the results show that a reduction of bone erosion markers occurs in the serum of MSD patients after radon spa therapy, but the systemic changes of factors involved in bone metabolism are not pronounced. However, anti-inflammatory and immune suppressive effects are suggested by the significantly altered systemic levels of the adipokine visfatin and Treg cells.

## Discussion

Our work was embedded in a large study (RAD-ON01 study), in which 103 patients suffering from MSDs have been enrolled; 100 of them were followed up by regular medical examinations for 30 weeks after treatment. Long-lasting pain reduction was observed for the majority of the patients (36). This is in good agreement with results from preceding studies on other pathologies in which analgesic effects and functional improvements after radon treatment have been shown [e.g., IMURA (39)].

In the frame of the RAD-ON01 study, further investigations performed in parallel with medical examinations were dedicated to unravel the cellular and molecular basis of the observed pain reduction and functional improvements. So far, detailed immune phenotyping on the blood samples from individual patients revealed a concomitant modulation of the peripheral immune cells (36). In the RAD-ON01 study that we present here, we set out to assess in a subset of patients markers of bone metabolism and related factors. We detected changes which are potentially related to bone metabolism, i.e., a decrease of collagen fragments (CTX-I, Figure 1A), a systemic decrease of the inflammatory factor visfatin (Figure 3A), and a shift in T cell subpopulations (Th17/Treg cells, Figure 4) following radon spa treatment. As these results have been obtained in a longitudinal study, we can demonstrate for the first time long-lasting pain relief after radon intervention in MSD patients occurring concomitantly with changes in the immune system and bone erosion.

Comparing our results of MSD patients with data on other treatment modalities reveals that CTX-I baseline levels and its 30% decrease after radon spa therapy (Figure 1A; Table S1 in Supplementary Material) matches well with the respective values of RA patients after anti-TNF-α therapy (40).

**Figure 5:**
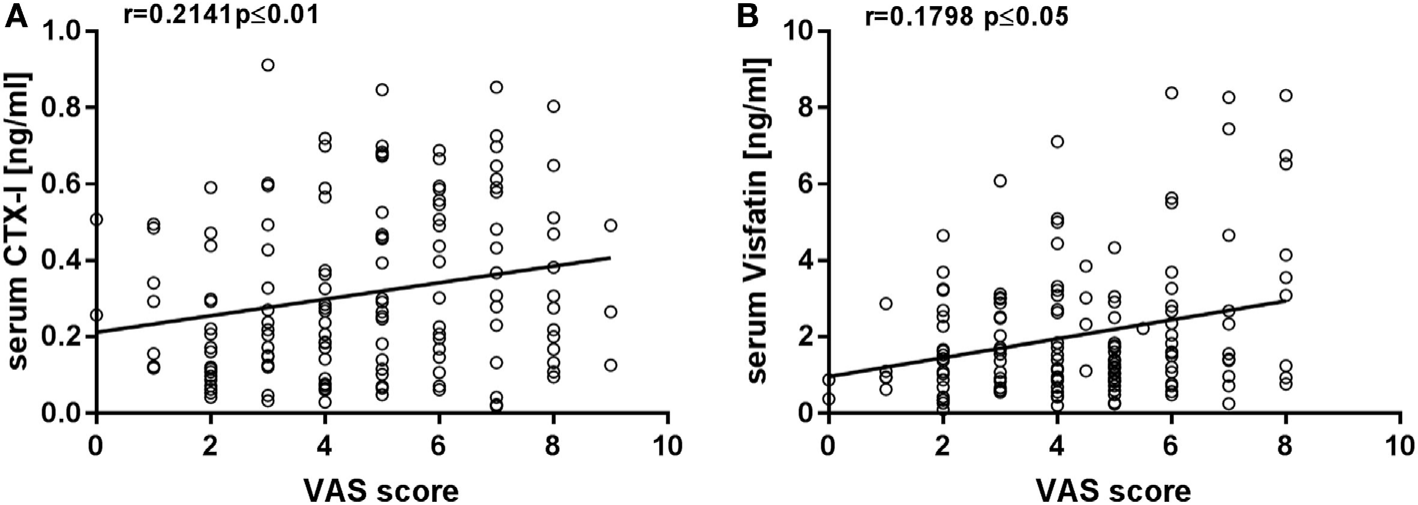
Correlation analysis between VAS pain score (0 = no pain, 10 = worst pain imaginable); data from a RAD-ON01 study published in (36) and CTX-I **(A)** or visfatin **(B)** in patients with MSD before and after radon treatment. *N* = 32, Spearman’s rank correlation, *P ≤ 0.5, **P ≤ 0.01.

Compiled results from prospective studies in osteoporosis patients and different treatment modalities showed a decrease of CTX serum levels between 10 and 80% (41). For bisphophonate treatment, a decrease of 63% was observed (42). To further investigate the relevance of the measured reduction of CTX-I levels in MSD patients, we have performed a correlation analysis between the CTX-I levels and the individual pain perception of the patients. We used data published in Ref. (36), where for the same patients pain perception was determined by visual analog scales (VAS; 0 = no pain, 10 = worst pain imaginable) as part of the regular medical examination (Figure 5A). The Spearman’s correlation coefficient was determined (*r* = 0.2141; *P* ≤ 0.01), indicating a positive correlation. This suggests a clear impact of radon spa treatment on bone metabolism, in line with the observed functional improvements in patients after the same type of treatment (36). Here, we also observe a small increase in cartilage attrition, a characteristic of early stage OA (Figure 1B). This is not consistent with the results obtained for CTX-I levels.

The importance of OPG and RANKL as molecular markers for bone formation and resorption, respectively, is well established (43, 44). The baseline levels of released protein in MSD patients in this study (Figure 2) are comparable to those published for AS and RA patients (45–47). However, data on serum levels of OPG and RANKL after radon exposure are scarce. Not for patients, but for individuals at risk for developing osteoporosis, a persistently increased ratio was reported following subjection to a combination of radon treatment and physical exercise (48). In other patient studies, OPG/RANKL was measured only before and directly after therapy. For AS patients, it was reported that the levels of both proteins are slightly modified and that this in turn results in an increase of the OPG/RANKL ratio (46). Similar changes, albeit more pronounced, were observed for RA patients but not for OA (49) patients. These results are slightly different from the results of the RAD-ON01 study revealing small and transient changes of OPG levels in MSD patients, but unchanged levels of RANKL (Figure 2; Figure S1A in Supplementary Material). No significant changes occurred in other markers indicating a calcitonin-mediated regulation of bone resorption (Figure S1D in Supplementary Material) or OCN- and BAP-mediated regulation of bone formation (Figures S1B,C in Supplementary Material). Therefore, we suggest that radon exposure does not lead to persistent systemic changes in the OPG/RANKL pathway in MSD patients. This is in good agreement with the unchanged levels of TNF-α, an inflammatory cytokine inducing osteoclastogenesis (50), which we measured in MSD patients after radon spa treatment (Figure S5 in Supplementary Material). However, an effect involving OPG/RANKL may be local and confined to sites of bone formation and resorption.

Adipokines are involved in the pathogenesis of RA and other autoimmune diseases (28, 51), but the specific influence of adipokines on bone metabolism in different pathologies, including OA, is less clear (52). Interestingly, adipokines are produced by cells of the adipose tissue, and adipose tissue displays a higher solubility for the lipophilic noble gas radon compared to water. Thus, we assume an accumulation of radon derived isotopes in infrapatellar fat pad of joints, bone marrow and in visceral fat. This has already been shown for fatty compounds (53); and our own unpublished observations support this view (A. Maier, GSI, personal communication). Hence, radon could modulate the release of adipokines by fat cells.

To test this hypothesis, we measured the level of adipokines in the frame of our study. The results revealed no significant changes for adiponectin, resistin, and leptin levels in the serum of MSD patients (Figure 3; Table S1 in Supplementary Material). In previous studies, pharmacological treatments however affected the levels of adiponectin and resistin, although the reported effects were not consistent. For example, a reduction of adiponectin levels has been shown in RA patients after a combined corticoid and anti-TNF-α therapy (32), whereas in other studies an increase was observed (54). Hence, at present, the effects of pharmacological treatments on some adipokines remain elusive, possibly related to an impact of the disease stage or metabolic alterations. In addition, the relation between high levels of the abovementioned adipokines and MSD are controversially discussed (52).

Importantly, for visfatin, high serum levels are reported for RA patients and correlate with several disease markers (52, 55). In the present study, we revealed that radon therapy causes a 50% reduction of the visfatin levels (Figure 3; Table S1 in Supplementary Material). This decrease is similar to the baseline levels reported for RA patients and the respective reduction found in some, albeit not in all studies after anti-TNF-α therapy (33, 34). To further assess the relevance of the reduction in visfatin levels for pain, we determined the Spearman’s correlation coefficient (*r* = 0.1798; *P* ≤ 0.05) using data from Ref. (36), indicating a positive correlation with pain perception (Figure 5B). This is in line with other studies showing an association of visfatin levels, pain, and joint damage (55, 56). We conclude that the decrease in visfatin levels and the concomitant lower pain perception in the radon-treated MSD patients shown in this study provide evidence for the role of visfatin in MSD, which can be targeted by a treatment with radiation.

However, in spite of an increased number of studies on adipokines (22, 57), it cannot be decided yet, if the decrease in visfatin levels elicited by radon or drug treatment is related to either bone resorption or to an impact on inflammation. The hypothesis of an impact of radon spa treatment on inflammatory processes is endorsed by a trend to an increase in immune suppressive and anti-inflammatory Treg cells that we detected by the intracellular marker FOXP3 (Figure 4). This is in line with the proposed role, which T cells play in the progression of OA and RA (29).

## Conclusion

We report here for a subset of MSD patients, enrolled in the RAD-ON01 study, a reduction of bone degradation, presumably related to an attenuation of inflammation, mediated by the adipokine visfatin and a changed ratio of the T cell subpopulations. The results are in line with pain reduction and systemic immune effects, i.e., a shift to anti-inflammatory or immune suppressive processes, observed in the frame of the RAD-ON01 study (36). However, the reduction of bone degradation was not reflected by a modified release of respective regulatory proteins, i.e., OPG/RANKL, in the serum of the patients. Therefore, further investigations on local cellular processes in inflamed joints after radon therapy are needed. It is noteworthy that with respect to radiotherapy of tumors, very low doses, as they might occur in the tumor surrounding, normal tissue, can induce an increase of Treg cells. In the scenario of a tumor therapy this may contribute to a tumor permissive microenvironment, and as such are a possible target for immune therapy (58).

## Ethics Statement

This study was carried out in accordance with the recommendations of the ethical review committee of the Bavarian State Chamber of Physicians (Bayerische Landesärztekammer, Munich, Germany, ethical approval BLAK #12131). All subjects gave written informed consent in accordance with the Declaration of Helsinki. The protocol was approved by the ethical review committee of the Bavarian State Chamber of Physicians.

## Author Contributions

AC and KS contributed equally to this work. GK, BF, UG, and CF: conception or AC, KS, BF, and UG, CF: design the work. AC, KS, PR, GK, and BF: acquisition and analysis, AC, KS, DK, GT, UG, and CF: interpretation of data for the work. All authors: drafting the work or revising it critically for important intellectual content; final approval of the version to be published; agreement to be accountable for all aspects of the work in ensuring that questions related to the accuracy or integrity of any part of the work are appropriately investigated and resolved.

## Conflict of Interest Statement

The authors declare that the research was conducted in the absence of any commercial or financial relationships that could be construed as a potential conflict of interest.

## Abbreviations

AS, ankylosing spondylitis; BAP, bone-specific alkaline phosphatase; COMP, cartilage oligomeric matrix protein; csDMARDS, conventional synthetic disease modifying drugs; CTX-I, collagen fragments type-I; NSAID, non-steroidal anti-inflammatory drugs; LDRT, low-dose radiation therapy; MSDs, musculoskeletal disorders; OA, osteoarthritis; OC, osteoclast; OCN, osteocalcin; OPG, osteoprotegerin; RA, rheumatoid arthritis; RANKL, receptor activator of nuclear factor kappa B (NFκB) ligand.
