# Supplementary material for: Decrease of Markers Related to Bone Erosion in Serum of Patients with Musculoskeletal Disorders after Serial Low-Dose Radon Spa Therapy
Source: Front Immunol. 2017 Jul 25;8:882. doi: 10.3389/fimmu.2017.00882 (PMC5524779; doi:10.3389/fimmu.2017.00882)
Supplement: Supplementary file 1 [file Data_Sheet_1.docx]

Supplementary Material

Decrease of markers related to bone erosion in serum of patients with musculoskeletal disorders after serial low-dose radon spa therapy

**A.Cucu^#1^, K.Shreder^#1^, D. Kraft^1^, P. Rühle^2^, G. Klein^3^, G. Thiel^4^, B. Frey^2^, U.Gaipl^2^, C.Fournier^1*^**

*** Correspondence:** c.fournier@gsi.de

Additional factors are indicative for changes in the balance of bone formation and degradation and have been measured in the serum of those patients, where enough material was available, and not in all 32 patients presented in the paper. We completed the assessment of RANKL by measuring an OPG-unbound form of RANKL (1), defined as “free” sRANKL. In addition, we investigated other factors, such as a bone-specific alkaline phosphatase (BAP) and osteocalcin (OCN), indicating the regulation of bone formation (2–4). Furthermore, we were interested to know whether increased levels of calcitonin contribute to the inhibition of OC activity, as calcitonin is a negative regulator of osteoclastogenesis (5,6).

The amount of free sRANKL was determined using ELISA obtained from Biomedica (Vienna, Austria). Bone-specific alkaline phosphatase (BAP) and osteocalcin (OCN) were measured with *in vitro* diagnostic applicable ELISA assays obtained from Immunodiagnostic Systems Ltd (Frankfurt/Main, Germany), calcitonin ELISA was purchased from BioVendor (Brno, Czech Republic). All measurements were carried out according to the manufacturer’s instructions.

As shown in Figure S1, none of the measured factors showed a significant change after radon spa treatment compared to baseline levels before therapy.

**Figure S1**

Figure S1: Effect of radon spa treatment on the levels of (A) free sRANKL, (B) bone-specific alkaline phosphatase (BAP), (C) osteocalcin (OCN) and (D) calcitonin in the serum of patients with MSDs, measured at indicated times before (0 weeks) and after the onset of the therapy (6-30 weeks). Boxplots show the median, Tukey whiskers (median ± 1.5 times interquartile range), mean (+) and outliers (•). N=16 for free sRANKL, N=23 for BAP, N=12 for OCN and N=15 for calcitonin.

In addition to the measurements of 32 patient samples presented in the paper, we show here the data set completed by measurements of additional patients. The data sets comprise a compilation of the results obtained in 32 patient samples, presented in Fig.1, 2 and 3, and additional patient samples, randomly chosen according to their availability.

Neither for CTX, COMP, OPG, nor for visfatin, adiponectin or resistin the results are changed by the increase in patients number (Fig. S2, S3).

**Figure S2**

Figure S2: Effect of radon spa treatment on the levels of (A) collagen fragments type-1 (CTX-1), (B) cartilage oligomeric matrix protein (COMP) and (C) osteoprotegerin (OPG) in the serum of patients with MSDs, measured at indicated times before (0 weeks) and after the onset of the therapy (6-30 weeks). Boxplots show the median, Tukey whiskers (median ± 1.5 times interquartile range), mean (+) and outliers (•). N=40 for free CTX-I, N=37 for COMP and N=34 for OPG. *P ≤ 0.05, ***P ≤ 0.001, CTX-I and COMP: Wilcoxon matched-paired signed rank test; OPG: two-tailed t- test. The data sets comprise compiled results (Fig.1 and 2, plus additional patient samples).

**Figure S3**

Figure S3: Effect of radon spa treatment on the levels of visfatin, adiponectin and resistin in serum of patients with MSDs. The concentration of appropriate adipokines was measured at the indicated weeks before (0 weeks) and after onset of the therapy (6-30 weeks). Boxplots show the median, Tukey whiskers (median ± 1.5 times interquartile range), mean (+) and outliers (•). N=44 for visfatin, N=39 for adiponectin and N=34 for resistin. *P ≤ 0.05, **P ≤ 0.01, ****P ≤ 0.0001, Wilcoxon matched-paired signed rank test. The data sets comprise compiled results (Fig.3, plus additional patient samples).

We further analyzed the levels of CTX, based on gender distribution and age of the patients (Fig. S4). We found that baseline levels are higher in female compared to male patients (Fig. S4 A), probably due to postmenopausal changes related to the mean age the patients. Both for male and female patients, radon spa treatment led to a decrease in serum levels of CTX. No correlation was found with the age of the patients (Fig. S4 B).

**Figure S4**

Figure S4: (A) Effect of radon spa treatment on the CTX-I levels in the serum of male (N=12) and female (N=28) MSD patients, measured at the indicated time points before (0 weeks) and after the onset of the treatment (6-30 weeks). Boxplots show the median, Tukey whiskers (median ± 1.5 times interquartile range), mean (+) and outliers (•). *P ≤ 0.05, **P ≤ 0.01, Wilcoxon matched-paired signed rank test. (B) Relation between CTX-I levels and age of the patients (Spearman correlation, P = 0.7451, r = 0.06194).

We measured in addition the serum levels of TNF-α, a major key player in chronic inflammation. The measurements were performed with Human TNF alpha ELISA Ready-SET-Go!® from eBioscience (Frankfurt/Main, Germany) according to manufacturer’s instructions. We did not detect significant changes comparing the TNF-α amount before and after radon spa treatment (Fig. S5).

**Figure S5**

Figure S5: Effect of radon spa treatment on TNFα levels in the serum of patients with MSDs, measured at indicated times before (0 weeks) and after the onset of the therapy (6-30 weeks). Boxplots show the median, Tukey whiskers (median ± 1.5 times interquartile range), mean (+) and outliers (•). N=12

Values obtained for all measured factors as presented in Fig 2A, 3B and Fig. S1, S2, S3, S5 are summarized in table 1

Table 1: Serum concentrations of factors related to bone metabolism and adipokines in MSD patients during and after radon spa treatment (before, 0 weeks and after the onset of the treatment, up to 30 weeks). Shown are the mean values within the range of the lowest and highest value and the number of analyzed patients.

**References**

1. Hofbauer LC, Heufelder AE. The role of osteoprotegerin and receptor activator of nuclear factor kappaB ligand in the pathogenesis and treatment of rheumatoid arthritis. Arthritis Rheum. 2001 Feb;44(2):253–9.

2. Patti A, Gennari L, Merlotti D, Dotta F, Nuti R. Endocrine actions of osteocalcin. Int J Endocrinol. 2013;2013:846480.

3. Hauschka PV, Wians FH. Osteocalcin-hydroxyapatite interaction in the extracellular organic matrix of bone. Anat Rec. 1989 Jun;224(2):180–8.

4. Baht GS, Hunter GK, Goldberg HA. Bone sialoprotein-collagen interaction promotes hydroxyapatite nucleation. Matrix Biol J Int Soc Matrix Biol. 2008 Sep;27(7):600–8.

5. Granholm S, Lundberg P, Lerner UH. Calcitonin inhibits osteoclast formation in mouse haematopoetic cells independently of transcriptional regulation by receptor activator of NF-{kappa}B and c-Fms. J Endocrinol. 2007 Dec;195(3):415–27.

6. Keller J, Catala-Lehnen P, Huebner AK, Jeschke A, Heckt T, Lueth A, et al. Calcitonin controls bone formation by inhibiting the release of sphingosine 1-phosphate from osteoclasts. Nat Commun. 2014;5:5215.
